# Ofloxacin as a Disruptor of Actin Aggresome “Hirano Bodies”: A Potential Repurposed Drug for the Treatment of Neurodegenerative Diseases

**DOI:** 10.3389/fnagi.2020.591579

**Published:** 2020-10-06

**Authors:** Samridhi Pathak, Haifa Parkar, Sarita Tripathi, Avinash Kale

**Affiliations:** School of Chemical Sciences, University of Mumbai - Department of Atomic Energy Center for Excellence in Basic Sciences, University of Mumbai, Vidyanagari Campus, Mumbai, India

**Keywords:** ofloxacin, neurodegenerative diseases, actin, repurposable drugs, biophysical studies, SEM, *in silico*

## Abstract

There is a growing number of aging populations that are more prone to the prevalence of neuropathological disorders. Two major diseases that show a late onset of the symptoms include Alzheimer’s disorder (AD) and Parkinson’s disorder (PD), which are causing an unexpected social and economic impact on the families. A large number of researches in the last decade have focused upon the role of amyloid precursor protein, Aβ-plaque, and intraneuronal neurofibrillary tangles (tau-proteins). However, there is very few understanding of actin-associated paracrystalline structures formed in the hippocampus region of the brain and are called Hirano bodies. These actin-rich inclusion bodies are known to modulate the synaptic plasticity and employ conspicuous effects on long-term potentiation and paired-pulse paradigms. Since the currently known drugs have very little effect in controlling the progression of these diseases, there is a need to develop therapeutic agents, which can have improved efficacy and bioavailability, and can transport across the blood–brain barrier. Moreover, finding novel targets involving compound screening is both laborious and is an expensive process in itself followed by equally tedious Food and Drug Administration (FDA) approval exercise. Finding alternative functions to the already existing FDA-approved molecules for reversing the progression of age-related proteinopathies is of utmost importance. In the current study, we decipher the role of a broad-spectrum general antibiotic (Ofloxacin) on actin polymerization dynamics using various biophysical techniques like right-angle light scattering, dynamic light scattering, circular dichroism spectrometry, isothermal titration calorimetry, scanning electron microscopy, etc. We have also performed *in silico* docking studies to deduce a plausible mechanism of the drug binding to the actin. We report that actin gets disrupted upon binding to Ofloxacin in a concentration-dependent manner. We have inferred that Ofloxacin, when attached to a drug delivery system, can act as a good candidate for the treatment of neuropathological diseases.

## Introduction

Neurodegenerative disorders (NDs) are the subset of brain disorders defined by the obliteration of neuronal cells resulting from the accumulation of protein aggregates (Perl et al., 1995). Deaths related to NDs are second-most around the globe and a prominent cause of disability worldwide (Feigin et al., 2019). An estimated 12 million Americans will be suffering from NDs by 2030, of which patients suffering from Alzheimer’s disease would be on top of the list (Chanfreau et al., 2005; Gammon, 2014). The biological factors that are known to cause NDs are attributed to oxidative stress, cytoskeletal protein aggregation, abnormal ubiquitination of protein, mitochondrial dysfunction, etc. (Trends and Disorders, 2018). Diseases like Alzheimer’s disorder (AD), Parkinson’s disease (PD), Huntington’s disease (HD), amyotrophic lateral sclerosis (ALS), frontal temporal dementia (FTD) falls under the category of NDs and a major contributor to the socioeconomic problems associated with it (Subramaniam, 2019). Various factors have been associated to be the causative agent behind NDs, but a prominent one is still far from the search. Depending on the type of NDs, it could be either familial or sporadic (Przedborski et al., 2003). The current treatment regime focuses on slowing the manifestations of the symptoms and providing temporary relief toward these symptoms, thus, causing a severe lack of procedure to slow the disease progression and eventual death (Trends and Disorders, 2018).

Cytoskeletal protein has a major role to play in neuronal functioning by providing flexibility and maintaining the neuronal circuit (Muñoz-Lasso et al., 2020b). Certain proteins that are widely studied for their role in NDs are prion, tau, β-amyloid, α-synuclein, and Huntington (Gitler et al., 2017). It is implicated that misfolding of these proteins leads to their aggregation in the neuronal cells resulting in neurodegeneration (Ross and Poirier, 2004; Santa-Mara et al., 2008; Goebel, 2009; Spears et al., 2014; Koistinen et al., 2017). However, the role of cytoskeletal protein actin in the manifestation of NDs have been far from understood. Actin is reported to be the driving force in controlling synaptic plasticity and maintaining the structural integrity of the synapses (Kim and Lisman, 1999; Luo, 2002; Fukazawa et al., 2003; Bourne and Harris, 2008; Okada and Soderling, 2009). It functions by changing its morphology in response to different types of neural activity (Svennberg, 2006; Gordon-Weeks and Fournier, 2014; Spence and Soderling, 2015; Szabó et al., 2016; Pelucchi et al., 2020). Defects in the regulation of actin protein is one of the contributing factors leading to neurological disorders (Kim and Lisman, 1999; Luo, 2002; Bourne and Harris, 2008; Okada and Soderling, 2009).

Actin cytoskeletal remodeling and rearrangement play an important role at different sites in the brain cells and different stages of brain activity (Kevenaar and Hoogenraad, 2015; Yamada and Kuba, 2016). In the axonal cells, actin occurs as a meshwork of branched filaments (Yamada and Kuba, 2016). During polarization of neurons, actin and its regulators control the assembly and disassembly of F-actin filament in order to regulate the axonal elongation and contribute to the formation of axonal filopodia (Kevenaar and Hoogenraad, 2015). Aberrations in the axonal cytoskeletal-dependent process lead to defects in axonal transport, outgrowth, targeting, and synapse functioning, which, in turn, is associated with ALS (Kevenaar and Hoogenraad, 2015). In the mature neurons, actin is associated with regulating the presynaptic functions such as conscripting and repositioning of a synaptic vesicle, maintaining exocytosis and endocytosis. Mis-regulated actin in mature neurons has been implicated in various mental illnesses such as intellectual disability and schizophrenia (Ogawa and Rasband, 2008). Actin polymerization dynamics has a major role in sustaining the morphology of the spine and is associated with long-term memory via activation of long-term potentiation (LTP) or long-term depression (LTD) of excitatory signal transmission (Kim and Lisman, 1999; Luo, 2002; Bourne and Harris, 2008; Okada and Soderling, 2009). Also, actin rods are involved in the progression of axonal sensory neuropathy: a condition where neurons are damaged as observed in frataxin-deficient dorsal root ganglion (DRG) neurons (Muñoz-Lasso et al., 2020a).

The presence of F-actin aggregates has also been found in Hirano bodies, which are one of the causes of AD (Sabo et al., 2001; Maselli et al., 2003; Melidone et al., 2011; Furgerson et al., 2012; Yao and Khan, 2013). Hirano bodies are cytoplasmic inclusion bodies, rod-shaped eosinophilic in nature. It was first found in the hippocampus region of the aged brain in patients suffering from ALS and PD (Goldman, 1983; Sabo et al., 2001; Maselli et al., 2003; Melidone et al., 2011; Furgerson et al., 2012; Yao and Khan, 2013). The protein aggregate was filamentous, paracrystalline in nature, and thin filaments of 6 mm in size were observed. Actin depolymerization factor (ADF)/cofilin, an actin-binding protein, which helps sever F-actin, is severely reduced under stress conditions thereby enhancing the formation of aggregates (Yang et al., 2010; Muñoz-Lasso et al., 2020a, b). This leads to the development of rod-like structures of actin filaments widely present in neurons of patients with AD and PD (Furukawa and Fechheimer, 1997; Yang et al., 2010).

Owing to the aforementioned problems associated with actin misfolding, dysregulation, and its subsequent aggregation, it can form a potential therapeutic target for neurological disorders. A previous study by Pathak et al. (2020) elucidates the effect of the tetracycline group of antibiotics on F-actin protein aggregate. It has paved a way to think toward the detection of targeted therapies against F-actin aggregates. Due to this, the use of Food and Drug Administration (FDA)-approved drugs could form a potential alternative to act against actin aggregates responsible for neurodegenerative and neurodevelopmental disorders. Drug repositioning has become one of the widely used approaches in recent times, as it is highly efficient, economical, and less vulnerable. This may have a major impact on the reduction of financial burden, the progression of the disease, and subsequently lower the time taken for the drug discovery (Eira et al., 2016; Pushpakom et al., 2019; Pathak et al., 2020).

Ofloxacin is a broad-spectrum antibiotic widely used to treat infections caused due to *Staphylococcus aureus*, *methicillin-resistant Staphylococcus aureus (MRSA)*, *Streptococcus* spp., *Enterococcus faecalis*, Enterobacteriaceae, and *Pseudomonas aeruginosa*. It comes under the class of fluoroquinolones and structurally, it is a tricyclic ring that has a methyl group being attached to the C-3 position (asymmetric carbon) on the oxazine ring (Morrissey et al., 1996). Owing to the safety of this drug, which has passed all the clinical trial phases, our study has identified it as a molecular entity with a new function. In the current study, we have investigated the role of Ofloxacin on F-actin aggregates using various biophysical techniques such as right-angle light scattering (RLS), dynamic light scattering (DLS), circular dichroism spectroscopy (CD), and kinetics study. The imaging studies using scanning electron microscopy (SEM) were also carried out, and the plausible mode of binding of Ofloxacin to F-actin protein was studied using isothermal titration calorimetry (ITC) and *in silico* studies. We have proposed a plausible mode of binding of Ofloxacin to actin and its subsequent disruption into the smaller oligomeric state. We thus propose that actin aggregates can be broken into smaller oligomers and be made available for new rounds of polymerization using Ofloxacin, if we have a proper drug delivery system in place, which is capable of crossing the blood–brain barrier (BBB).

## Materials and Methods

### Purification and Characterization of Actin

All chemicals used for the experiment were procured from S.D Fine (Mumbai, India) except for DTT and Ofloxacin (PubChem CID: 4583), which were procured from Sigma Aldrich (Mumbai, India). Acetone powder from pig thigh muscle (*Sus scrofa domesticus*) was prepared following a standard protocol (Solomon et al., 2010). Actin was purified using the method developed by Spudich et al. in the year 1971 for rabbit skeletal muscle (Solomon et al., 2010) with minor modifications. In brief, acetone powder was homogenized in G-actin buffer (GB) (composition: 2 mM Tris–HCl, 0.2 mM ATP, 0.5 mM DTT, and 0.2 mM CaCl_2_) followed by treatment with polymerization buffer (PB: Composition: 800 mM KCl and 5 mM MgCl_2_). It was then kept for overnight polymerization with constant stirring at 4°C, followed by ultracentrifugation at 75,000 rpm for 90 min. The ultracentrifuge used was a tabletop model from Beckman Coulter XP-100 at Bombay College of Pharmacy (BCP), Mumbai, India. Purified polymer actin (F-actin) was characterized on MALDI-TOF (Bruker Daltonik GmbH) (Chavan et al., 2016) at the Advanced Centre for Treatment, Research, and Education in Cancer (ACTREC), Navi Mumbai, India. In order to exchange the solvent system from PB to either GB or water, the F-actin pellet was resuspended in the respective solvent and was dialyzed against the desired solvent system for 72 h at 4°C with buffer change every 12 h.

### Light-Scattering Measurements

**FIGURE 1:**
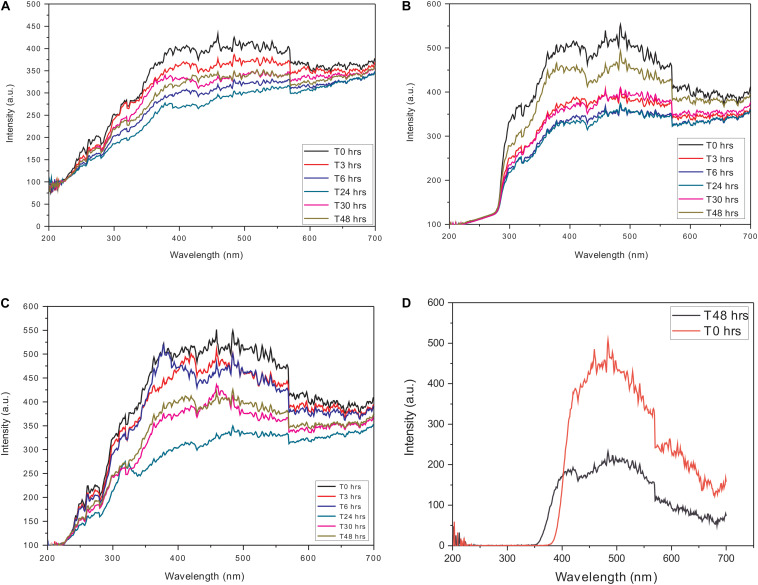
Constant wavelength synchronous analysis (CWSF) profile for actin control in **(A)** polymerization buffer (PB), **(B)** G-actin buffer (GB), **(C)** water, and **(D)** Ofloxacin control in PB. Black color represents CWSF at 0 h, red color represents CWSF at 3 h, blue color represents CWSF at 6 h, green color represents CWSF at 24 h, pink color represents CWSF at 30 h, and the cyan color represents CWSF at T48 h.

To avoid any interference in the scattering analysis, independent constant wavelength synchronous fluorescence (CWSF) measurements were carried out on actin and Ofloxacin in the polymerization buffer system. All the aforementioned compounds were procured from Himedia (Mumbai, India) and Sigma Aldrich. All these measurements were carried out on Cary Eclipse Fluorescence Spectrophotometer from Agilent technologies with the following parameters while keeping the difference between excitation and emission wavelength (Δλ) at zero: excitation wavelength: 200–700 nm, emission wavelength: 200–700 nm, excitation slit width: 5, emission slit width: 5, excitation filter: auto, emission filter: auto, temperature: room temperature, time points: T0, T3, T6, T24, T48, and T72 h. It should be noted that we have shown data only up to 48 h for clarity purposes. The concentration of actin was fixed at 3 μM, whereas, for Ofloxacin, measurements were carried out for the highest concentration of 3,000 μM to ensure that there is no concentration-dependent aggregation of the compound. The synchronous curves are depicted in Figure 1. Likewise, measurements were also carried out for water and G-actin buffer systems.

Based on the findings of the synchronous measurements, all the scattering experiments were carried out using the following parameters: excitation wavelength: 350 nm, emission wavelength: 350 nm, excitation slit width: 5, emission slit width: 5, excitation filter: auto, emission filter: auto. The concentration of actin protein was kept constant at 3 μM and was incubated with varying concentrations (3, 30, 150, 300, 750, 1,500, and 3,000 μM) of Ofloxacin. Scattering measurements were carried out at the following time points: T0, T3, T6, T12, T18, T24, T36, T48, and T72 h. These measurements were done in the three different solvent systems, namely, PB, GB, and water.

### Dynamic Light Scattering Studies

The effect of Ofloxacin on the oligomeric state and heterogeneity of F-actin was measured using DLS. The experiments were performed on Malvern Panalytical (United Kingdom) at the Department of Biophysics, University of Mumbai, India. The position of the attenuator was set at 4.65, which was determined automatically based on the size and the concentration of the actin polymer in association with the respective compounds. The samples were taken in a polystyrene disposable sizing cuvette, and folded capillary zeta cell was used for the measurement of zeta potential. F-actin was prepared in PB buffer, and the concentration was kept constant at 3 μM. The concentration of Ofloxacin was maintained at 300 μM (compound ratio to actin was kept at 1:100). At least two measurements were carried out for each of the interactions between F-actin and the drug. A detection angle of 90° was used for the measurement of the size. The analysis was carried out on a zeta sizer Nano-ZS90 DLS system at 25°C, equipped with red (633 nm) laser and avalanche photodiode detector (quantum efficiency > 50% at 633 nm). The software associated with this DLS machine was Dispersion Technology Software (DTS) version 7.0, which was used to analyze the z-average hydrodynamic radius, intensity distribution, volume distribution, polydispersity index (PDI), autocorrelation function, and zeta potential (Liu et al., 2012).

### Circular Dichroism Spectroscopy

The changes in the secondary structure of F-actin in the three different solvent systems, namely, PB, GB, and water were monitored using CD spectroscopy. All measurements were carried out between 260 and 200 nm using a JASCO-J-815 spectropolarimeter at the National Centre for Cell Sciences (NCCS), Pune, India. The protein was prepared in the respective buffer, and the concentration was fixed at 5 μM. The ratio of protein:drug Ofloxacin was taken as 1:5 (25 μM), 1:10 (50 μm), 1:20 (100 μM) 1:50 (250 μM), and 1:100 (500 μM). The parameters used in the experiment were as follows: the bandwidth was maintained at 1.00 nm, and the cell of 1 mm path length was used. The data were presented as mean residue ellipticity [θ] in deg cm^2^ dmol^–1^, which is defined by equation 1 below:

(1) [θ]=CD/10×n×1×Cp)

where CD is in millidegree, n is the number of amino acid residues, 1 is the path length of the cell in cm, and Cp is the molar concentration of the protein (Sonavane et al., 2017). The K2D2 (Perez-Iratxeta and Andrade-Navarro, 2008) software was used, and further analysis of the data was done using CAPITO (Wiedemann et al., 2013).

### Imagining Studies Using Scanning Electron Microscopy

The SEM analysis was carried out on a ZEISS microscope using the following parameters: EHT (3 kV) and signal (SE2). It should be noted that all the imaging assays were performed in GB buffer, and water as PB buffer was high in salt concentration, thereby, limiting us for imaging in PB buffer. SEM imaging was carried out at the Tata Institute of Fundamental Research (TIFR), Mumbai.

### Kinetic Measurements for Actin Aggregation

The rate of depolymerization of F-actin in the presence and absence of Ofloxacin was performed using different concentrations of drugs ranging from 3 to 90 μM. The concentration of actin was fixed at 3 μM and was prepared in PB. The effect of the drug on the depolymerization kinetics of actin was measured using RLS on spectrofluorimeter (Agilent technology) (Borana et al., 2014). These measurements were performed at room temperature with excitation and emission wavelengths fixed at 350 nm. In each case, the slit width for both excitation and emission was kept at 5 nm. The measurements were carried out for 30 min to ensure the attainment of saturation points for the respective compounds. The above parameter for the kinetic analysis was derived based upon our initial synchronous experiments with the offset value of zero (Borana et al., 2014).

All the kinetic traces were adequately fit to a single exponential decay curve as defined by the following equation (Equation 2):

(2) $$y=y\,0+A\,2\times \exp\left(-\frac{x}{t\,1}\right)$$

where A2 stands for the amplitude, y0 stands for the maximum degradation/depolymerization, and t1 stands for the time constant. Depolymerization rate constant K_*app*_ was apparently calculated as the inverse of the apparent rate constant.

$$K_{a\,p\,p}=1/t$$

Since all of the traces showed nearly to be a two-phased reaction, the amplitude of the first phase reaction was calculated by the following equation (Equation 3):

(3) $$A\,1=y_{\max}-A\,2$$

where *y*_*max*_ is the RLS value in the absence of drugs.

### Isothermal Titration Calorimetric Measurements

Isothermal titration calorimetric (ITC) experiments were carried out to understand the association constant (Ka), thermodynamic parameters of binding (ΔH), entropy (ΔS), and the stoichiometry (n) of the interaction of the drug with F-actin. The protein was prepared in PB buffer and kept for dialysis for 72 h at 4°C on a magnetic stirrer. All the measurements were carried out on a Malvern MicroCal ITC 200. The following parameters were used for the analysis: the total number of injections for Ofloxacin was 19, cell temperature was kept at 25°C, reference power was kept at 10, the initial delay was 180, cell concentration (F-actin): 0.067 mM, syringe concentration for Ofloxacin was 1.34 mM (20×).

### *In silico* Data Analysis

Actin interaction with that of Ofloxacin was carried out in order to predict the site of binding of the drug to the actin oligomer (Hexamer). For all our analyses, we had used the ADP bound actin monomer (PDB ID: 1J6Z) (Otterbein et al., 2001). The structure was carefully observed and was duly corrected for incomplete residual side chains using the simple mutate function of the WinCoot tool (Crystallographic Object-Oriented Toolkit) (Emsley et al., 2010; Debreczeni and Emsley, 2012). This modified monomer was used to prepare a hexametric polymer using the cryo-EM structure, 3J0S as a template molecule using the SSM superpose function of the WinCoot. Subsequently, the hexamer was energy minimized using the online Gromacs Minimizer 5.0 tool (Abraham et al., 2015). The 3D structure of Ofloxacin was downloaded from PubChem (CID: 4583) and saved as an SDF file. This SDF file was then converted to PDB using the Discovery Studio 2019 Client (BIOVIA Discovery Studio Visualizer).

AutoDockTools-1.5.6 was used to prepare the “.pdbqt” files for both actin polymer and Ofloxacin. AutoDock Vina 4.2.6. was used for carrying out the docking studies of the actin with the drug utilizing the standard search parameters (Trott and Olson, 2010). Obtained results were analyzed using Pymol as a visualization tool.

## Results

### Purification and Characterization of Actin

Our sequence analysis showed that human actin was having more than 90% similarity to that of pig actin. Actin being highly conserved across the higher eukaryotes, the pig (*S. scrofa domesticus*) thigh muscle was used for the purification of actin. In order to purify actin, acetone powder was prepared from pig thigh muscle and stored at −80°C. Actin was purified using the protocol as mentioned and modified by Pathak et al. (2020) in batches for all our assays. The purified actin had a mass of 42 kDa as observed in our SDS page analysis, which was also confirmed by excising the aforementioned band from the gel and subjecting it to mass spectrometric analysis.

### Constant Wavelength Synchronous Analysis

In order to perform RLS analysis for the actin–Ofloxacin interaction, it was important for us to know the parameters for the same. We performed CWSF for both the drug molecule (Ofloxacin) as well as actin in three different buffer systems viz: PB, GB, and Water. It was observed in our results (Figures 1A–C) that actin in all the three buffer systems shows a very high scattering from 250 to 700 nm up to 48 h. Ofloxacin has negligible scattering observed up to 390 nm beyond, which starts showing a very high scattering as observed in Figure 1D. Owing to our CWSF data, we performed all our right-angle scattering analysis at a wavelength of around 350 nm, as actin in all the three buffer systems showed significant scattering, while Ofloxacin did not show any scattering. This would, hence, avoid any interference from the drug while collecting the data for right-angle scattering.

### Right-Angle Light Scattering Measurements

**FIGURE 2:**
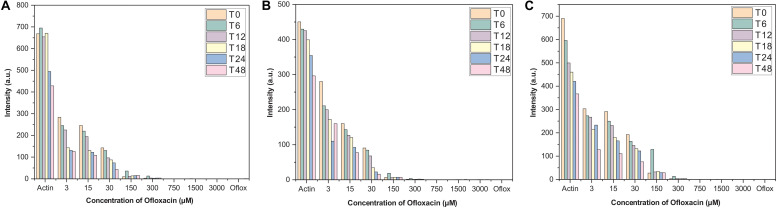
Right-angle light scattering (RLS) profile for actin control and actin treated with Ofloxacin in **(A)** PB, **(B)** GB, and **(C)** water. The orange bar stands for actin aggregates at 0 h, green for 6 h, purple for 12 h, yellow for 18 h, blue for 24 h, and pink for 48 h.

Actin–Ofloxacin interaction was studied using RLS at the different concentrations for up to 48 h in the three aforementioned buffer systems. We observed that actin control in buffer systems showed a very high scattering of around 700 nm in PB and water and around 450 nm in GB. This high intensity was directly proportional to the size of the aggregate present in our control systems. Both PB and water has polymerized filamentous actin as well as aggregated actin, while GB has oligomeric actin present in them indicating the three different morphological states of the protein as found in the *in vivo* system. Also, there was very little drop in the measured intensity of the actin controls with respect to time, which was indicative of actin’s intrinsic property to polymerize and depolymerize. Upon treatment with different concentrations of Ofloxacin as observed in Figures 2A–C, there was a proportional drop in the scattering intensity as the function of its concentration as well as the time of treatment. At higher concentrations from 30 to 3,000 μM, the scattering intensity is very low in all the three buffer systems indicating that Ofloxacin upon interaction with either the polymerized actin, higher oligomeric actin, or aggregated actin gets disintegrated to smaller oligomeric and subsequently to monomeric actin. This interaction is also shown to be irreversible as there was no rise in the scattering intensity even after 48–96 h.

### Dynamic Light Scattering for Actin Compound Interaction Studies

**FIGURE 3:**
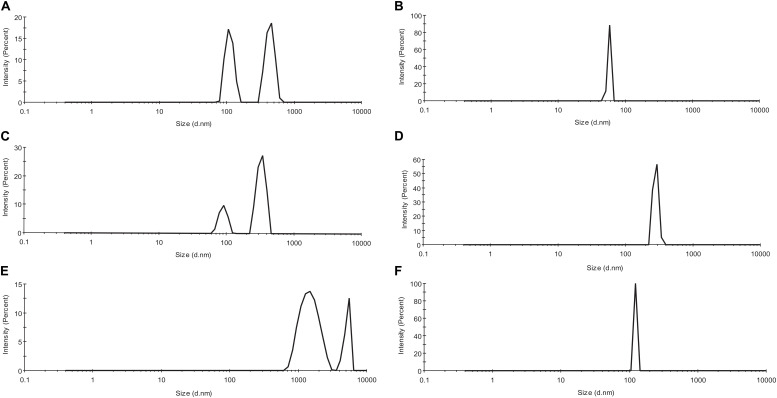
Profile for dynamic light scattering (DLS) scattering of **(A)** untreated actin in PB; **(B)** actin treated with Ofloxacin in PB; **(C)** untreated actin control in GB; **(D)** actin treated with Ofloxacin in GB; **(E)** untreated actin control in water; and **(F)** treated actin with Ofloxacin at 300 μM.

As observed from Figures 3A,C,E, actin control in the three solvent systems *viz*, PB, GB, and water shows the presence of a heterogeneous population of actin aggregates, which varies in size. The two peaks vary in the size of aggregated actin present in each of the three solvent systems. In PB, the size was deciphered to be around 90–150 d nm for the first peak, from 350 to 800 d nm. In GB, the size of the two peaks has been deduced to be 80–120 and 300–400 d nm while in water, the size of the two peaks was more than 1,000 d nm. This size variance and heterogeneity of the peaks show that actin control has a different morphology in the three different solvent systems. However, upon treatment with Ofloxacin, actin in PB, GB, and water were disintegrated into a homogenous population of smaller monomeric/oligomeric state actin as observed in Figures 3B,D,F respectively. The size of the homogenous peaks was deduced to be 70–80 d nm in PB, 450–500 d nm in GB, and 100–120 d nm in water.

### CD Spectrophotometric Analysis of the Actin Compound Interaction Studies

**FIGURE 4:**
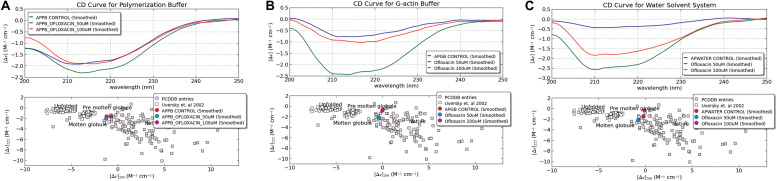
CD Spectroscopic data analyzed for actin control and actin treated with Ofloxacin using CAPITO software. **(A)** PB; **(B)** GB; and **(C)** water. The blue curve is representative of actin control, whereas the green curve is representative of actin treated with Ofloxacin at 50 μM, and the red curve is representative of actin treated with Ofloxacin at 100 μM in the respective buffer.

We also performed circular dichroism spectroscopic analysis (CD) for both untreated actin control and treated actin with Ofloxacin in PB, GB, and water, respectively. The CD data obtained in mdeg was analyzed using the CAPITO software. As observed in Figures 4A–C, actin control in PB, GB, and water shows the curve to have a single peak dip at 212 nm indicative of the presence of more polymeric, filamentous, or aggregated structures. However, upon treatment with Ofloxacin, actin in all the three respective buffer system shows the change in the curve profile to contain more α-helical structure closer to globular actin monomer with two dips observed at 211 and 220 nm.

Our CAPITO analysis as observed in Figures 4A–C also suggests the compactness of the actin protein upon treatment with different concentrations of Ofloxacin either as globule, molten globule, premolten globule, or unfolded protein. Untreated actin in PB exists as molten globule, which is indicative of compact partially folded conformation with near-native compression, whereas in GB and water, it exists as a globular structure. As soon as we treat actin in the PB buffer system with Ofloxacin at 50 and 100 μM, a concentration-dependent change in the structural content of the actin was observed. At 50 μM, the actin oligomer lies between the conformation of the molten globule and globular protein indicating the presence of a larger oligomeric actin with increasingly exposed hydrophobic residues (Perera et al., 2016). However, as soon as the concentration is increased to 100 μM, actin, which is further disrupted to either small oligomers or monomers, has a compact near-native structure with substantial α-helical content and more organized tertiary structure. Similarly, when actin in GB was treated with Ofloxacin, there was a structural change observed with respect to the concentration of the drug molecule. At a lower concentration of 50 μM, actin is disintegrated into smaller oligomers and monomers prevailing as a molten globular protein with partially exposed hydrophobic residues rich in α-helix. However, as soon as the concentration is increased, the partially folded actin protein is switched back to the more globular structure. In water, where the actin exists as an amorphous aggregate, the structural premise of the protein is more globular. As soon as it is treated with Ofloxacin at a concentration of 50 μM, it occurs as a molten globule and changes to a more globular structure at a higher concentration of 100 μM. This structural change in actin in water is quite contrary to that observed in actin treated in PB and GB.

**FIGURE 5:**
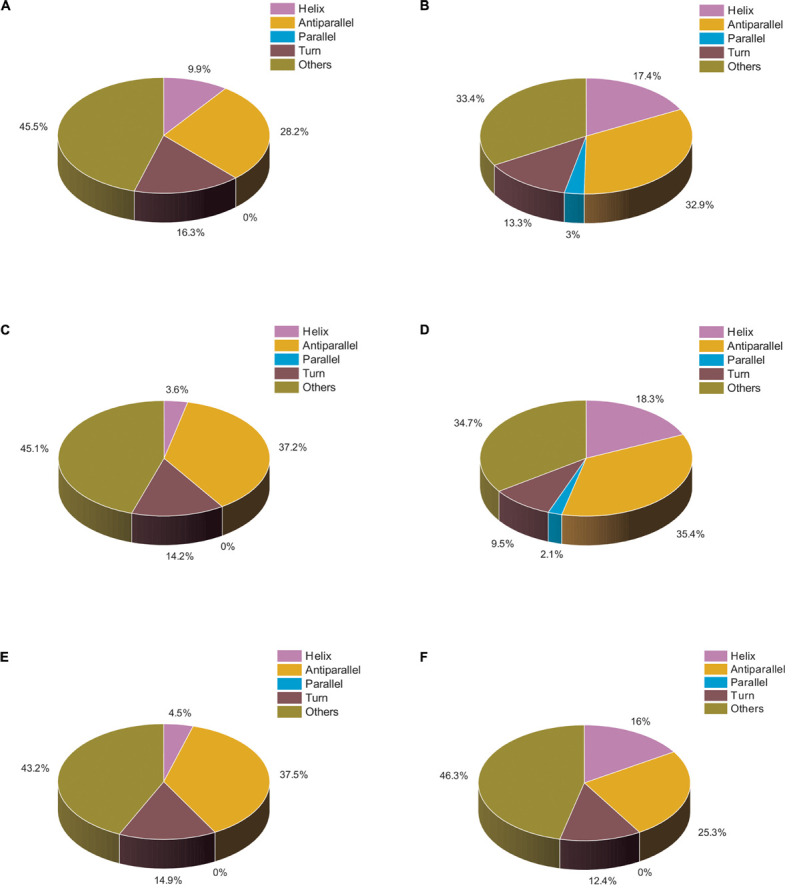
Pie chart representation of the structural distribution of treated and untreated actin. **(A)** Actin control in PB. **(B)** Actin treated with Ofloxacin (50 μM) in PB. **(C)** Actin control in GB. **(D)** Actin treated with Ofloxacin (50 μM) in GB. **(E)** Actin control in water. **(F)** actin treated with Ofloxacin (50 μM) in water.

We also analyzed the same set of data using BESTSEL software to calculate the percentage of α-helical and β-structure present in untreated and treated actin. Our BESTSEL analysis as observed in Figure 5A, is that actin control in polymerization buffer has 9.9% of α-helix, 26.2 β-sheet, 16.3% turns, and 45.5% other non-organized structures. However, upon treatment with Ofloxacin in PB as observed in Figure 5B, the content of α-helix was increased to 17.4%, β-sheet was increased to 35.3%, while turns and other unorganized structures were reduced to 13.3 and 33.4%, respectively. Similarly, for the G-actin buffer, it was observed in Figures 5C,D that actin control had the presence of α-helix of around 3.6%, β-sheet, around 37.2%, turn, 14.2%, and other structures, 45.1%. However, the treated actin had an α-helix of around 18.3%, β-sheet 37.6%, turn, 9.5%, and other structures, 34.7%. Also, for actin control in water as observed in Figure 5E, we observed α-helix to be around 4.5%, β-sheet, 37.5%, turn, 14.9%, and other structures, 43.2%. However, upon treatment, the structural change was reported to comprise 16% α-helix, 25.3% β-sheet, 12.4% turn, and 46.3% other unorganized structure as observed in Figure 5F. Our result of the BESTSEL analysis indicates that posttreatment of polymerized/aggregated actin resulted in actin enriched in α-helical content which is likely to be closer to the globular actin molecule.

### SEM Imaging for the Actin Compound Interaction

**FIGURE 6:**
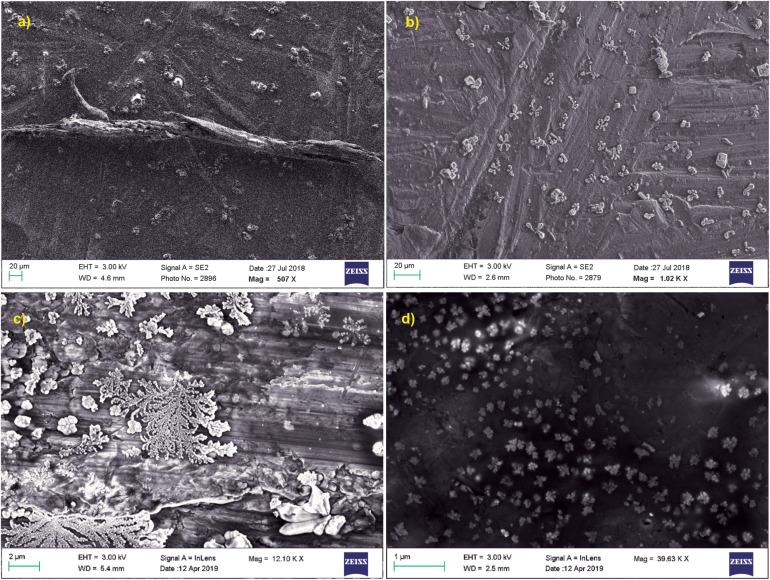
SEM images for actin **(a)** dialyzed in G-actin buffer 20 μm; **(b)** treated with Ofloxacin in G-actin buffer 20 μm; **(c)** dialyzed in water 2 μM; and **(d)** treated with Ofloxacin in water 2 μM.

Scanning electron microscopy was performed for both treated and untreated actin in GB as well as water. PB was not used for these imaging studies due to the presence of high salt concentration in the buffer (800 mM KCl), which gave rise to more salt crystals to be observed in the SEM image. As observed in Figures 6a,c, untreated actin dialyzed against GB and water showed the presence of filamentous actin as well as amorphous actin aggregates, which when treated with Ofloxacin gets broken down to a smaller oligomeric protein as observed in Figures 6b,d.

### Kinetic Analysis of Actin Depolymerization

**FIGURE 7:**
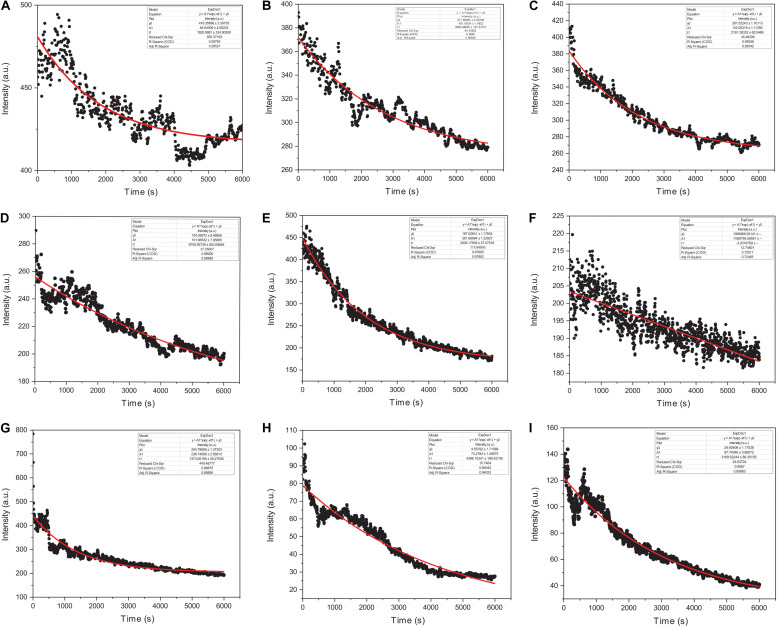
Graphical representation of actin polymerization dynamics in the presence and absence of Ofloxacin at different concentrations observed in PB. **(A)** Actin control; **(B)** treated actin at 3 μM; **(C)** treated actin at 6 μM; **(D)** treated actin at 9 μM; **(E)** treated actin at 15 μM; **(F)** treated actin at 30 μM; **(G)** treated actin at 45 μM; **(H)** treated actin at 60 μM; **(I)** treated actin at 90 μM. The red straight line indicates the single exponential curve fit analysis while the black dots represent the RLS intensity recorded every 5 s of actin–Ofloxacin interaction.

We monitored the kinetic parameters for actin interactions with Ofloxacin in polymerization buffer as a function of right-angle scattering, which was measured every 5 s for up to 100 min. It is a known fact that right-angle scattering is proportionate to the size of the molecule. As observed in Figures 7A–I, the graph shows actin control in PB and the effect of Ofloxacin with its varying concentrations, which was analyzed and fit using a single exponential decay function of the kinetics. Three different parameters were reported through this fitting viz: (1) kagg, (2) amplitude (A2), and (3) y0. kagg stands for rate constant representing the speed of the reaction upon the interaction of actin polymer and Ofloxacin. A2 stands for amplitude indicating the amount of smaller actin oligomers formed upon interaction with Ofloxacin. y0 stands for the extent to which the depolymerization has occurred upon interaction with the drug at the infinite (∞) time. The *R*^2^ value for the single exponential decay fit was calculated to be between 0.97 and 0.72 as analyzed for varying concentrations of Ofloxacin interaction with actin.

**FIGURE 8:**
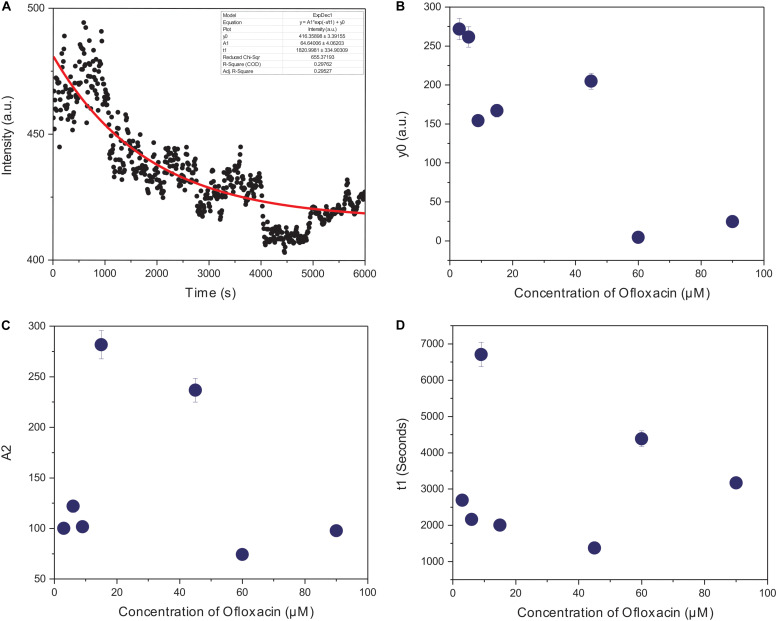
Graphical representation of representative kinetic profiles in PB for actin treated with Ofloxacin: **(A)** Actin control; **(B)** extent of disintegration (y0); **(C)** amplitude (A2); and **(D)** time constant (t1).

The kinetics of polymerized F-actin in PB was measured for 100 min of its intrinsic polymerization and depolymerization dynamics that occur at the barbed end and the pointed end, respectively. We observed (Figures 7A, 8A) that actin control in polymerization buffer is quite stable and does not disintegrate substantially with the values for amplitude observed as 64.64 a.u., y0, 416.35 a.u., and t1, 1,820.99 s. However, upon treatment with Ofloxacin with a stoichiometric ratio of 1:1 as observed in Figure 7B, the amplitude was calculated as 100.12 a.u., y0 was calculated as 271.80 a.u., and t1 was calculated as 2,693.98 s. With increasing concentration, a tremendous decrease in the intensity of the amplitude was observed indicating that Ofloxacin works to break down the highly aggregated actin into smaller oligomers. This process is concentration dependent. It was observed in our analysis that increasing concentration of Ofloxacin led to the decrease in the time required for breaking down the highly aggregated actin to smaller oligomers as well as the increase in the number of smaller oligomers formed. We also report the mode of interaction of the actin polymer with that of Ofloxacin, which follows a two-phased reaction. The first phase is quite faster than the second phase wherein an intermediate product is formed before the formation of the final product. The amplitude of the first phase (A1) can be calculated using Eq. (3) and is concentration dependent, while the second phase is not dependent upon the concentration of the Ofloxacin treatment.

Furthermore, we plotted the amplitude for the second phase reaction (A2), the time constant (t1), and y0 against the concentration of Ofloxacin. A graph of the extent of disintegration (y0) against the concentration of Ofloxacin shows (Figure 8B) that as the concentration of the drug molecule increases, there is also an increase in the disintegrated product. We observed (Figure 8C) that the amount of the smaller oligomer formed is highest at a very high concentration of around 90 μM. Our graphical analysis for rate constant shows that the speed of interaction of Ofloxacin with that of actin polymer and its subsequent breakdown increases with the increase in the concentration of Ofloxacin as observed in Figure 8D.

### Isothermal Titration Calorimetry Analysis for Actin Compound Interaction

**FIGURE 9:**
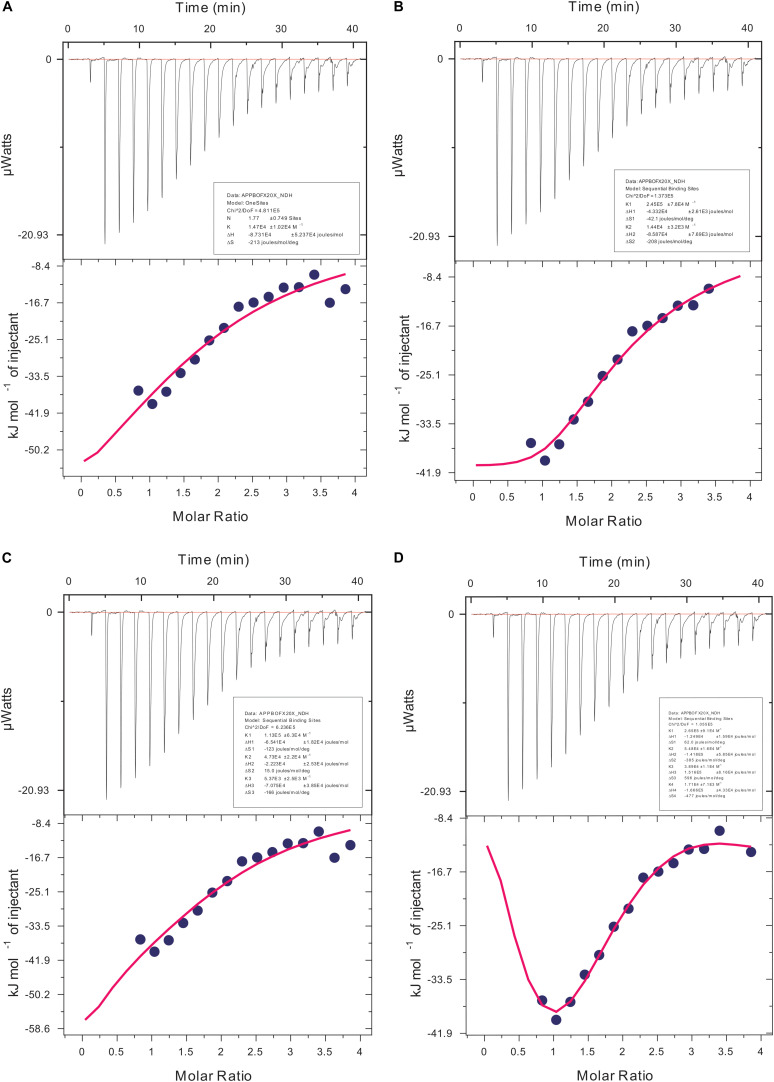
Isothermal calorimetric profile for actin aggregates treated with Ofloxacin in PB. **(A)** Model: one-site binding; **(B)** model: sequential two-site binding; **(C)** model: sequential three-site binding; and **(D)** model: sequential four-site binding.

We carried out our interaction study of actin polymer and Ofloxacin using ITC in order to deduce the thermodynamic parameters for the reaction as well as the mode of binding. Figures 9A–D show the ITC profile for the actin aggregates interacting with Ofloxacin in polymerization buffer analyzed and tailored using a different model system. The upper panel represents the endothermic heat pulse with the first injection of around 0.4 μl followed by 18 injections of 2 μl each of 1.34 mM Ofloxacin into the actin aggregate solution of 0.067 mM. The lower panel illustrates the integrated heat data indicative of the differential binding curve that was fit with using one-site binding, two-site sequential binding, three-site sequential binding, and four-site sequential binding model system. Although all the fit for the ITC data as seen in Table 1 shows binding to the actin of Ofloxacin strongly, the best fit was observed for the two-site sequential binding with the chi-square value of 1.373E5. The values of ΔH, ΔS, and K have been reported for all the four-binding models in Table 1. All of our binding fit shows that the reaction is both enthalpically as well as entropically driven. Two-site sequential binding of the drug molecule to the actin filament is enthalpically driven at both the sites of binding. This specifies that actin polymer/aggregate disruption upon binding of Ofloxacin is quite spontaneous and exothermic. Our data is indicative that Ofloxacin might be binding the actin polymer at multiple sites; however, two major sites on the actin polymer would be more favorable for Ofloxacin binding than the rest of the other binding sites.

**TABLE 1:** Thermodynamic parameters obtained using isothermal titration calorimetry (ITC) for Ofloxacin.

Parameters	One-site binding	Sequential two-site binding	Sequential three-site binding	Sequential four-site binding
Chi^∧^2/DoF	4.811 E5	1.373E5	6.236E5	1.055E5
K1	1.47E4 ± 1.02E4 M^–1^	2.45E5 ± 7.8E4 M^–1^	1.13E5 ± 6.3E4 M^–1^	2.65E5 ± 9.1E4 M^–1^
ΔH1	−8.731E4 ± 5.237E4 J/mol	−4.332E4 ± 2.61E3 J/mol	−6.541E4 ± 1.82E4 J/mol	−1.249E4 ± 1.59E4 J/mol
ΔS1	−213 J/mol/deg	−42.1 J/mol/deg	−123 J/mol/deg	62.0 J/mol/deg
K2		1.44E4 ± 3.2E3 M^–1^	4.73E4 ± 2.2E4 M^–1^	5.48E4 ± 1.6E4 M^–1^
ΔH2		−8.587E4 ± 7.69E3 J/mol	−2.223E4 ± 2.53E4 J/mol	−1.418E5 ± 5.85E4 J/mol
ΔS2		−208 J/mol/deg	15.0 J/mol/deg	−385 J/mol/deg
K3			5.37E3 ± 2.5E3 M^–1^	3.89E4 ± 1.1E4 M^–1^
ΔH3			−7.075E4 ± 3.85E4 J/mol	1.516E5 ± 8.16E4 J/mol
ΔS3			−166 J/mol/deg	596 J/mol/deg
K4				1.71E4 ± 7.1E3 M^–1^
ΔH4				−1.666E5 ± 4.33E4 J/mol
ΔS4				−477 J/mol/deg
	Enthalpically driven	Enthalpically driven	Enthalpically driven	Enthalpically and entropically driven

### *In silico* Data Analysis

**FIGURE 10:**
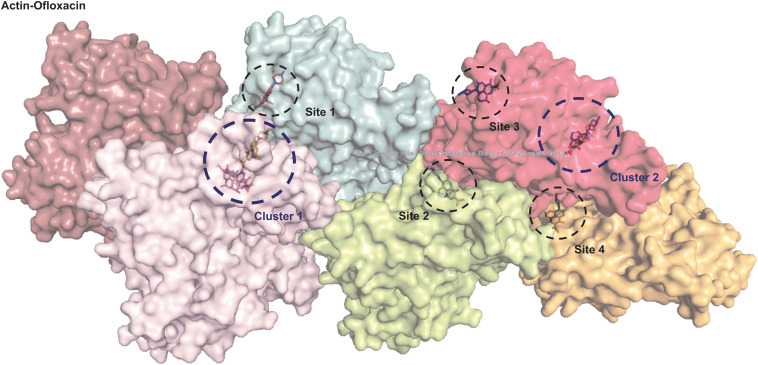
Actin polymer interaction with Ofloxacin. Hexamer actin. ChainA is shown salmon, ChainB in purple, ChainC in cyan, ChainD in lemon, ChainE in pink, and ChainF in orange.

We performed autodocking studies for hexameric actin interaction with Ofloxacin in order to deduce the possible mode of binding of the drug molecule to that of the filament. We observed in Figure 10 that most of the clusters of the Ofloxacin drug bind to the lateral interface of the actin timer. Various previous studies have suggested that actin during nucleation forms a rigid nucleus known for producing a critical concentration to promote the process of elongation of the filament (Cooper and Schafer, 2000; Vavylonis et al., 2005; Bugyi and Carlier, 2010; Blanchoin et al., 2014; Cell Signaling, 2014; De La Cruz and Gardel, 2015; Galkin et al., 2015; Coutts and La Thangue, 2016; Carlier and Shekhar, 2017). Our docking studies suggest that Ofloxacin tries to either inhibit by binding to the lateral interface as observed for Cluster 1, Site 2, Site 3, and Site 4 or disrupts the already formed nuclei in the system. It has also been observed in our data that the drug molecule is also getting associated with SD-2 of actin, which is rich in coiled coil as is prevalent in Cluster 2. It is an already known fact that these SD-2 of the actin monomer are responsible for the major conformational changes that drive the dynamics of actin polymerization (Blanchoin et al., 2014; Galkin et al., 2015; Carlier and Shekhar, 2017). We speculate that actin might be undergoing major conformational change upon Ofloxacin binding at SD-2, thereby, disintegrating the actin molecule. This data is quite in consensus with our ITC where the thermodynamics of the interaction reaction supports two-site sequential binding of the drug to actin.

## Discussion

Ofloxacin is one of the widely used broad-spectrum fluoroquinolone antibiotic used against several bacterial infections such as bronchitis, pneumonia, chlamydia, gonorrhea, skin infections, urinary tract infections, and infections of the prostate (Administration, n.d.; Kern et al., 1987; Eron and Gentry, 1992). Drug repositioning is a new approach that has been emerging recently for the treatment of various diseases as any lead molecule under a *de novo* drug discovery program, which takes around 10–15 years to come into the market and probably has a success rate of less than 10%. It has been a known fact that the FDA has approved different molecules against 400 human proteins (Pillaiyar et al., 2020). These proteins are classified under the umbrella of enzymes, transporters, G protein-coupled receptors (GPCRs), cluster of differentiation (CD) markers, voltage-gated ion channels, and nuclear receptors (Pillaiyar et al., 2020). Actin is one of the globular proteins with an ATPase binding cleft and an intrinsic property to polymerize (Oda et al., 2009; McKayed and Simpson, 2013). This polymerization dynamics that has been regulated by various actin-binding protein forms the driving force for many cellular processes such as cellular motility, cellular niche formation, and transport of biological molecules (Shekhar et al., 2016; Carlier and Shekhar, 2017). It has been observed that aberrations in the actin polymerization–depolymerization cycle lead to aggregation of the actin molecule leading to various diseased conditions (Lambrechts et al., 2004; Fletcher and Mullins, 2010; Muñoz-Lasso et al., 2020b). The unregulated actin dynamics has been implicated into the various neuropathological conditions, which manifest with the aging process among humans (Lambrechts et al., 2004; Gourlay and Ayscough, 2005; Goebel, 2009; Yao and Khan, 2012).

These neuropathological conditions are difficult to treat and lead to a decline in the quality of life. Thus, there is a widespread need for molecules with low cell toxicity to be identified for its ability to control and monitor cytoskeletal protein especially actin whose pathology has been implicated in arising cases of neurological apathies. Our current study emphasizes upon identifying a molecule, which can depolymerize the actin aggregates to smaller oligomers and prevent subsequent neuronal cell death that arises due to these aggregate formations. We utilized various biophysical techniques to study the role of Ofloxacin, a broad-spectrum antibiotic with a very low level of cell toxicity on actin polymerization–depolymerization dynamics. We also utilized *in silico* docking study in order to understand the mechanism of binding of Ofloxacin to actin and its subsequent role in breaking down the highly amorphous or polymerized actin to smaller aggregates.

Owing to the fact that human actin is closer to pig (*S. scrofa*) actin, we purified actin from the acetone powder of pig thigh muscle. The protein was purified upon several polymerization and depolymerization cycles in PB buffer rich in salt concentration and GB buffer, which has the presence of a reducing agent and ATP. The purified protein was isolated and separated at 42-kDa molecular weight on the SDS page. It was also confirmed using the excised band for mass spectrometric analysis to be actin. This protein was then used for further interaction studies with the drug Ofloxacin. We performed constant wavelength synchronous analysis to observe for the scattering profile for the three morphological states of actin in PB, GB, and water and the drug molecule in PB. This data was measured for up to 48 h. The measured data for the actin in three different buffers had the presence of high scattering intensity from 250 to 700 nm indicating toward the fact that all three morphological states of the actin remain in the filamentous, oligomeric, and aggregated state. However, when we did the same analysis for the drug, it was observed that actin shows negligible scattering up to 395 nm beyond, which starts self-aggregating resulting in a very high scattering intensity from 400 to 700 nm. Hence, for our further right-angle scattering measurement, we used 350 nm. Furthermore, we carried out a right-angle scattering measurement for actin–Ofloxacin interaction with varying concentrations of the drug molecule up to 48 h. We observed through our RLS measurement that actin controls in all the three buffer systems, which was having very high scattering intensity due to the presence of a higher molecular weight aggregate that gets disrupted to a smaller oligomeric size with low scattering intensity as the function of the concentration of Ofloxacin. This binding and disruption in all the three buffer systems were irreversible as no increase in the scattering intensity was observed posttreatment even after the 48 h of incubation.

We performed dynamic light measurement in order to deduce the particle size obtained posttreatment with Ofloxacin. It was observed that the heterogenous peak representing variations in shape and size of the actin aggregate in three different solvent systems was transformed to a homogenous peak of a smaller size indicating the breakdown of the actin polymer/aggregate to a smaller oligomeric or monomeric state. The structural change in the actin morphology pre- and posttreatment was analyzed using the CD spectrometric measurement, the data for which was collected in mdeg. The data for the untreated actin control as well as the treated actin in the three buffer systems were analyzed both manually using two different software viz: CAPITO (Wiedemann et al., 2013) and BESTSEL (Micsonai et al., 2018). The information obtained through our analysis indicated an increase in the structural population of the α-helix in treated actin compared to their untreated counterparts. The oligomers/monomers obtained posttreatment had a structural component closer to the content of globular actin, which is also rich in α-helix (Pathak et al., 2020). Actin in PB is more polymeric or filamentous with highly exposed hydrophobic residues and has near-native compactness, while actin in GB is more globular with native compactness of the protein and substantially a higher α-helix structure. This distinct feature of GB is due to the fact that this buffer is capable of disrupting the actin aggregate/polymer into a smaller oligomeric size of the actin. At a lower concentration of Ofloxacin, around 25 μM, we do not observe much change in the structural content from that of the treated actin. Thus, we choose to have the concentration of the drug molecule with a ratio of 1:10 and above. One of the important findings of our CD data using our CAPITO analysis was that treated actin in PB and GB brings about a concentration-dependent change from molten globule to that of the more globular structure. On the contrary, actin in water, which occurs as an amorphous aggregate, was seen to be occurring as a globular structure. Treated actin at 50 μM occurs as a molten globular structure with a more exposed hydrophobic structure with near-native compactness (Perera et al., 2016). As soon as the concentration is increased to 100 μM. actin changes from a molten globular structure to a more globular structure. This change, which is typical of water, is because of the amorphous aggregate that has a more unorganized structure, which is more spherical and compact, with relatively less exposed hydrophobic structure and a detectable tertiary structure. However, at a lower concentration of Ofloxacin, actin is disrupted as a more linear polymeric or large oligomeric secondary structure occurring as a molten globule with near-native compactness. At the increased concentration of 100 μM, actin is disrupted to monomeric or lower oligomeric structure rich in α-helix with near-native compactness and less exposed hydrophobic residues. Although we tried to increase the concentration of Ofloxacin with a ratio up to 1:50, it leads to the saturation of the CD detector.

We then also perform imaging studies to observe for the morphological difference in the treated and untreated actin. Control actin dialyzed against GB and water shows the presence of filamentous actin as well as amorphous actin aggregate. However, upon treatment with Ofloxacin, this highly filamentous as well as amorphous aggregate is converted to the morphology of smaller oligomers and monomers. We also performed the kinetic study for the actin–Ofloxacin interaction as a function of right-angle scattering measured against the varying concentrations of Ofloxacin. We tried to deduce three basic parameters from our study viz, y0, which represents the extent to which disintegration occurs, the time constant (t1), which is the time taken to break down and stabilize the reaction, as well as amplitude (A2), which represents the number of lower oligomers formed posttreatment with actin in polymerization buffer. Our analyzed data revealed that actin itself undergoes constant recycling of polymerization and depolymerization as a result of its intrinsic property as was observed for the actin control in PB. However, upon the treatment, we deduced that the amount of actin oligomeric content (A2) increased with an increase in the concentration of Ofloxacin, thereby, decreasing the content of highly aggregated actin in the system with each increasing concentration. We also see that actin polymer/aggregate upon treatment with Ofloxacin follows a two-phased reaction of which the first phase of the reaction remains tremendously fast compared to the second phase, which is relatively very slow. We observed that an intermediate product is formed before the formation of the end product in our kinetic analysis. The time required to stabilize the reaction was as short as 30 min, although our measurement was carried out up to 100 min and more. Most of the interaction was found to occur in the first phase, which is concentration dependent beyond which, as we enter the second phase post the intermediate product formation, actin disruption is independent of the concentration of Ofloxacin.

**FIGURE 11:**
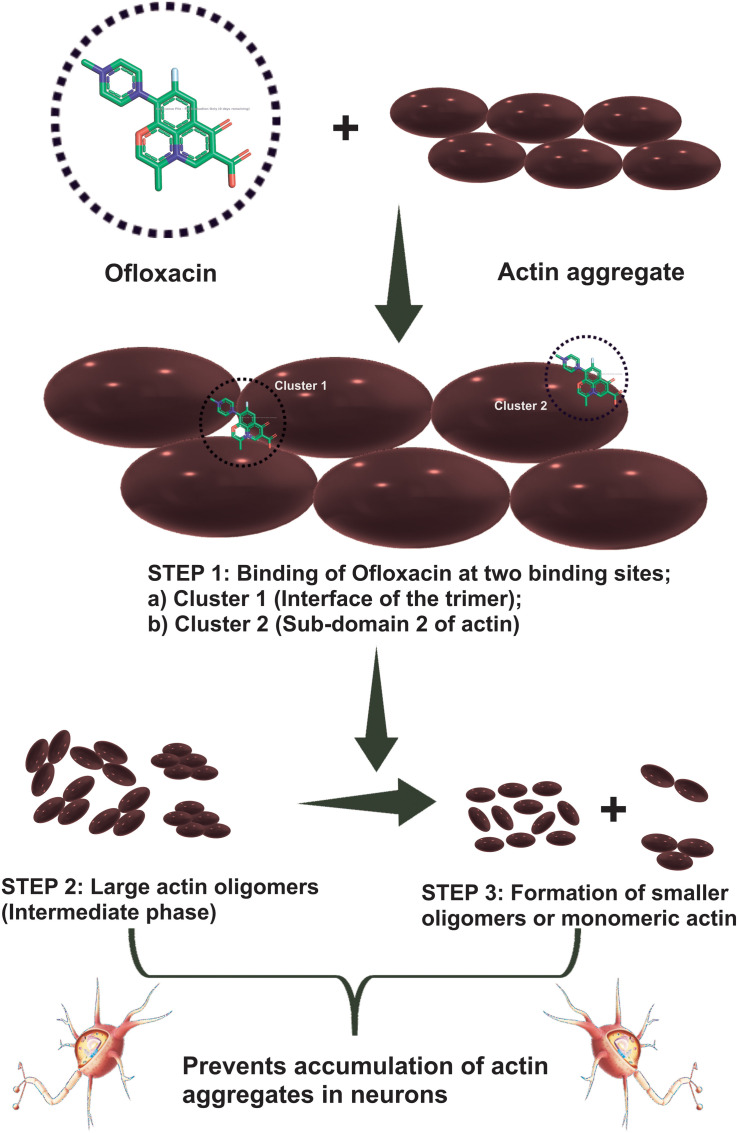
Cartoon representation of the mechanism of action of Ofloxacin on actin aggregates. Actin aggregates upon treatment with Ofloxacin result in binding at two sites on actin. Large oligomers are formed in the intermediate phase followed by the formation of smaller-sized oligomers of monomeric actin.

We then studied our interaction of actin–Ofloxacin in PB using ITC to deduce the thermodynamic parameters for the reaction and its mode of binding. Although our result shows binding for one site and multiple site sequential binding, the best fit was observed for two-site binding with low error values indicating that there are two major sites on actin polymer/aggregate, which favors the binding of Ofloxacin and subsequent disintegration of the actin molecule to a lower oligomeric size. We also observed that, on both of these sites, the interaction is enthalpically as well as entropically driven. Our *in silico* data shows two prevalent sites for binding of Ofloxacin to that of actin hexamer. These sites include the lateral interface, which is important for actin monomer interaction to form the nuclei, and the other site is near SD-2. We speculate that actin might undergo conformation change in its three-dimensional lattice upon Ofloxacin binding at SD-2 as well as inhibits the interaction of the actin monomer at the interface, which are responsible for nuclei formation as shown in Figure 11. This data agrees with our ITC data, which show a preferential mode of binding to the two-site sequential binding. The plausible mechanism that we have deduced is that Ofloxacin binds the actin at the aforementioned sites viz, cluster 1 and cluster 2, thereby, bringing about conformational change and destabilizing the larger aggregates. This is followed by the disintegration of large actin aggregates into the oligomeric structure. The accumulation of smaller nuclei or monomeric actin might prevent the obliteration of neuronal cells due to the inclusion bodies of actin.

## Conclusion

In the current study, Ofloxacin, which is a widely used broad-spectrum antibiotic for various bacterial infections, is elucidated as a potential candidate for drug repurposing. We studied actin aggregate as a significant therapeutic target site to treat various neurodegenerative as well as neurodevelopmental disorders. In order to study the role that Ofloxacin plays on these protein molecules, we purified actin from the pig thigh muscle (*S. scrofa domesticus*) in three different solvent systems. The three solvent systems, namely, PB, GB, and water mimic the *in vivo* morphology of the actin protein inside a human cell. These result in the actin being purified as a long filamentous polymer when dialyzed against PB, as smaller oligomers when dialyzed against GB, whereas in water, it formed amorphous aggregates. Pig thigh muscle was used as it is highly homologous with that of human actin with a 95% identity. Throughout the initial high-throughput screening of Ofloxacin on different morphologies of actin, we observed that the drug molecule was indeed disrupting large actin molecules.

In order to understand the actual role of Ofloxacin on actin aggregates, we performed CWSF analysis. During this assay, we observed that actin in all the three buffer systems shows higher scattering from 250 to 700 nm for 48–72 h without any drops. This indicates that actin remains as a large molecular-sized polymer, oligomer, or amorphous aggregate for a long period of time. We then performed the same assay for the drug up to 72 h, which resulted in Ofloxacin showing scattering post 400 nm wavelength. In order to avoid any major interference from the drug molecule during our RLS measurement, we used a wavelength of 350 nm as the scattering of the drug up to 390 nm remains negligible. During the right-angle scattering, we observed that there was a concentration-dependent drop in the scattering intensity of the actin molecule in all the three solvent systems. Since this drop in the intensity was observed up until 72 h, we concluded that Ofloxacin causes an irreversible disruption of larger actin aggregates. We then performed DLS, which resulted in the convergence of heterogenous peaks of actin aggregates in PB, GB, and water to a homologous peak of smaller sizes indicating the change in the actin morphology and abundance of either smaller actin oligomers or monomers.

We also performed CD spectroscopic analysis in all the three-buffer systems, which indicates a shift of a highly polymeric structure of untreated actin into an actin rich in α-helix posttreatment. This analysis leads us to conclude that differential actin aggregate changed to globular actin. In order to observe the morphological changes in actin, we also performed SEM analysis. Through our data, we reported that the disintegration of actin occurred in both GB and water upon treatment with Ofloxacin. Our kinetic data analysis of Ofloxacin interaction with that of actin polymer in polymerization buffer indicates that the disruption of larger actin aggregates followed a two-phase reaction. During the first phase of the reaction, which is much faster than the second phase, a large molecule of actin aggregate is formed into a smaller actin nuclei, which is further disrupted into a smaller oligomer or monomeric actin. The second phase of the reaction, which is relatively slow, does not show much change in activity upon the increase in the concentration of Ofloxacin. We thus concluded that only the first phase of the reaction, which forms the intermediate product of actin aggregates, is concentration dependent. Isothermal calorimetry data suggested that the best binding occurred with two set sequential bindings with the interaction being enthalpically and entropically driven. The interaction was further studied using *in silico* analysis of actin–drug association. It was understood from the auto-dock data that Ofloxacin binds to actin at two major sites viz, Sub-domain 2 as well as at the interface of the actin nuclei. Ofloxacin was supposedly found to interfere with both the longitudinal as well as lateral interactions between two associating actin monomers.

Through our biophysical and mechanistic understanding of actin–Ofloxacin interaction, we suggest that the drug could be used as a potential candidate against the prognosis and treatment of several neurodegenerative as well as a neurodevelopmental disorders. In order to make it to the market, much further work in the area of drug dosage and drug delivery of this molecule is required. A molecular vehicle, either organic or nanomaterial, capable of encapsulating the drug as well as crossing the BBB would prove to be beneficial in targeting the dysregulated actin aggregate formed in the neuronal cells.

## Data Availability Statement

All datasets presented in this study are included in the article/supplementary material.

## Author Contributions

SP executed the experiment, analyzed the data, and wrote the manuscript. HP executed the experiment and wrote the manuscript. ST executed the experiments. AK conceptualized the work, analyzed the data, and wrote the manuscript. All authors contributed to the article and approved the submitted version.

## Conflict of Interest

The authors declare that the research was conducted in the absence of any commercial or financial relationships that could be construed as a potential conflict of interest.
